# Data from brain activity during visual working memory replicates the correlation between contralateral delay activity and memory capacity

**DOI:** 10.1016/j.dib.2019.105042

**Published:** 2019-12-23

**Authors:** Mario Villena-González, Iván Rubio-Venegas, Vladimir López

**Affiliations:** a Escuela de Psicología, Pontificia Universidad Católica de Chile, Santiago, CP: 7820436, Chile; b Laboratorio de Neurociencia Cognitiva y Social, Facultad de Psicología, Universidad Diego Portales, Santiago, Chile

**Keywords:** Working memory, Memory capacity, Event related potential, Contralateral delay activity, Memory load, Change detection task

## Abstract

This article provides data from statistical analysis of event-related brain potentials (ERPs) and behavioural performance from 23 participants during a working memory task. Specifically, we used the change detection task from Vogel 2004, using the same timing but a modified size and distance between stimuli. Contralateral delay activity (CDA) was calculated from posterior parieto-occipital electrodes and then it was compared between conditions with different memory load (one, two and four items). Working memory capacity (WMC) was calculated from behavioural data using the formula developed by Pashler (1988). Correlation was performed between WMC and the CDA amplitude difference (from two to four items). The correlation replicates the results from the original paper of Vogel 2004 [1], even though some parameters are different from the original design.

**Table:** Specifications Table

Subject	Cognitive Neuroscience
Specific subject area	Neurophysiology of the Visuo-Spatial Working Memory
Type of data	Raw dataset Text file Graph Figure Table
How data were acquired	Electroencephalography (EEG) Biosemi active two 64 electrodes
Data format	Raw (BDF file) Analysed Filtered
Parameters for data collection	ERP was calculated time-locked to the memory array appearance, for three different memory load conditions; one item, two items and four items.
Description of data collection	Continuous EEG data was recorded during the experiment and data were off-line resampled to 1024 Hz, re-referenced to mastoids, filtered between 0.01 and 20 Hz and epoched for each experimental condition. Event-related potential (ERP) was calculated from contralateral and ipsilateral hemisphere at the posterior parieto-occipital electrodes. Subtraction between contralateral and ipsilateral signal was performed in order to obtain contralateral delay activity (CDA)
Data source location	Institution: Pontifical Catholic University of Chile City/Town/Region: Santiago Country: Chile
Data accessibility	Repository name: Mendeley Data Data identification number: 10.17632/j2v7btchdy.1 Direct URL to data: https://doi.org/10.17632/j2v7btchdy.1

**Table:** 

**Value of the Data** • These data are useful because they show that even by changing some experimental parameters, the results from an important research in the field of working memory remain consistent and replicable • Researchers in the field of neurophysiology of memory can benefit from these data because provides further confirmation on the brain potentials related to memory load and ensure reliability of the results regardless of physical parameters of stimuli. • These data could be useful to complement the discussion about the relationship between ERP amplitude measurements and behavior, but also to encourage design modifications in order to explore other features of the phenomenon of working memory • Since replication is an essential issue in science, we believe these data are valuable because provide support and information about reliability of results in the field of cognitive neuroscience

## 1 Data description

**Fig. 1:**
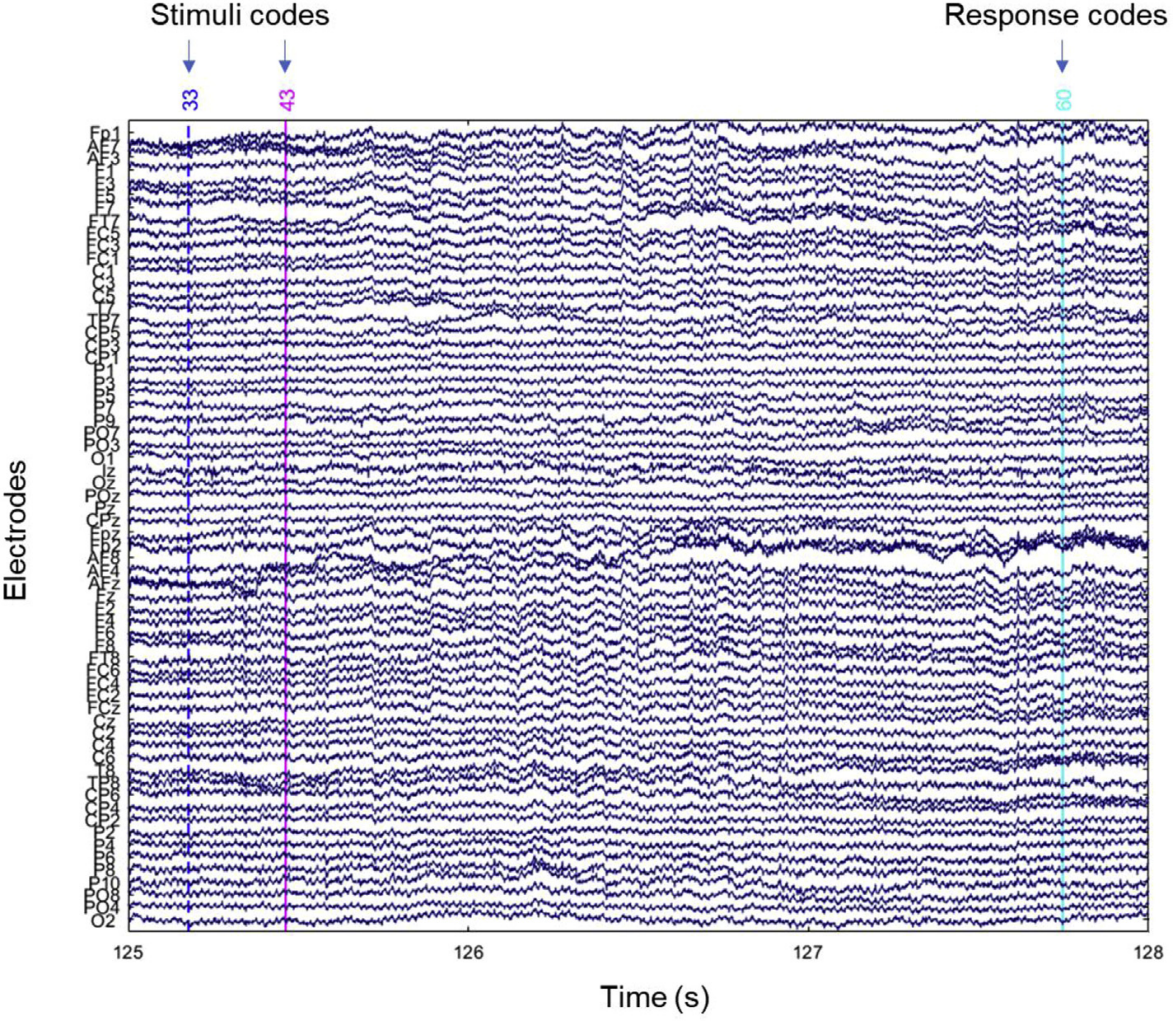
**Visualization of a sample trial from the raw file.** The first stimuli code is the arrow cue (irrelevant for further EEG analysis), the second one is for the memory array and the last one is the response code indicating correct or incorrect answers.

Dataset from Mendeley repository contains EEG raw data files from 23 participants while they performed a change detection task used in Vogel 2004 [1]. The task involved three different memory loads (one, two and four items). This data files can be found in BDF format, which can be visualized using MATLAB software (The Mathworks, Inc.), EEGLAB toolbox [2] and the data import extension BIOSIG. Fig. 1 shows an example of the visualization for one trial of the raw file. Details of the specific stimuli coding can be found in Table 1.

**Table 1:** Details of the specific stimuli coding for memory array and participants responses.

	Memory array codes		Participant responses	
	Left cued stimuli	Right cued stimuli	Correct answer	Incorrect answer
One item	11 or 12	10 or 13	60	61
Two items	21 or 22	20 or 23		
Three items	41 or 42	40 or 43		

**Fig. 2:**
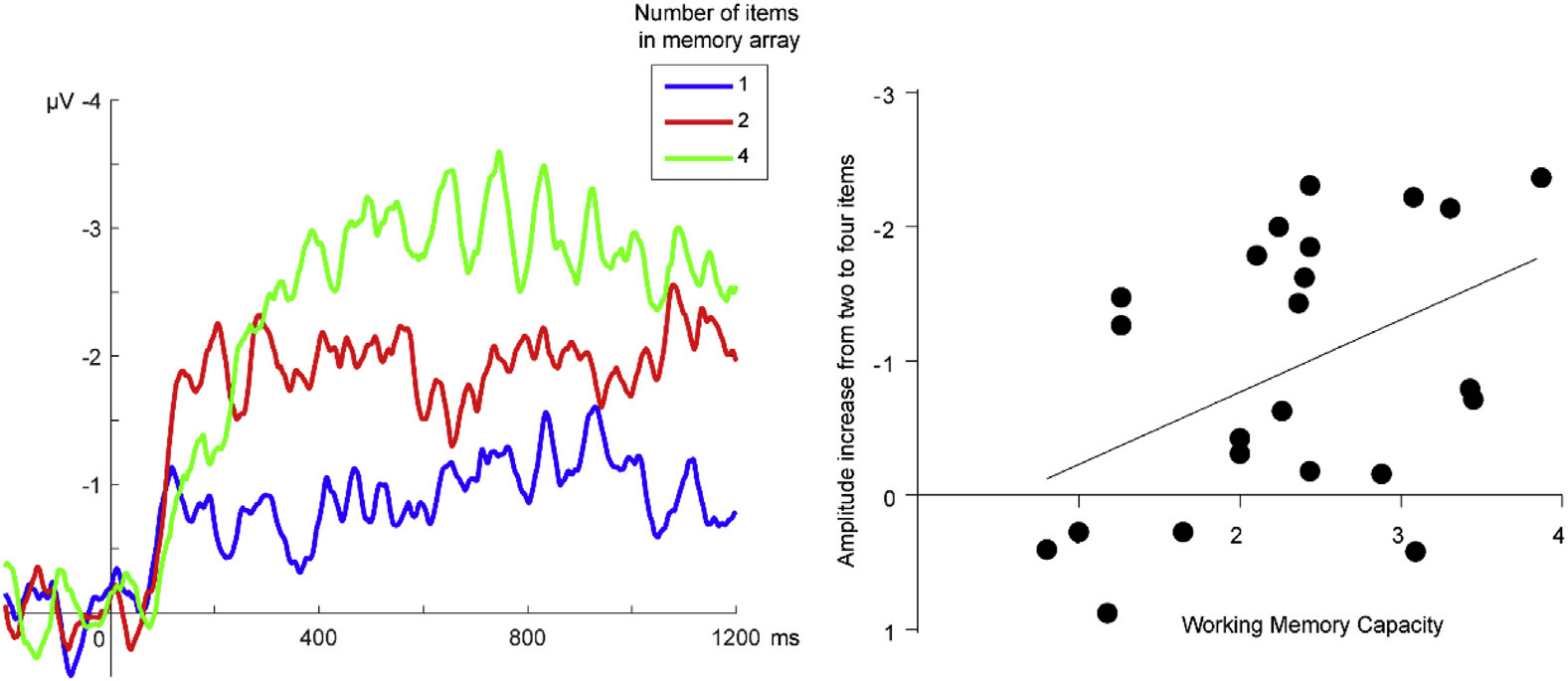
**Contralateral delay activity (CDA) correlates with Working Memory Capacity (WMC)**. Waveform of CDA for different number of items during memory retention (left). Correlation between the amplitude increase between two and four items and the WMC across participants (right).

Data shown in the present article is from the above-mentioned dataset, but after all the processing described in the following methods section (EEG Data pre-processing, ERP calculation and Statistics). In the section “Behavioral and electrophysiological data”, the total accuracy for the task and working memory capacity (WMC) can be found in the text, as the average across participants with the corresponding standard deviation. The EEG activity associated with memory retention known as Contralateral-delay activity (CDA) can be seen in Fig. 2 (left). This waveform is shown for three different number of items.

Statistics from Pearson correlation test can also be found in the text. This Correlation was calculated for the increase in amplitude of CDA between two and four items and WMC. This correlation plot can be found in the Fig. 2 (right). We also provide the statistics for correlation between the magnitude of CDA increase between one and four items and WMC.

## 2 Experimental design, materials, and methods

### 2.1 Participants

Twenty-three volunteers (15 women) were recruited for the study (mean age = 26.17, SD = 6.69). All participants had normal or corrected-to-normal vision and reported no color-vision deficiency. Participants had no history of drug abuse, neurological or psychiatric conditions. The protocol was approved by the Ethics Committee of Pontificia Universidad Católica de Chile. All subjects gave written informed consent in accordance with the Declaration of Helsinki. All experiments were performed at the Laboratory of experimental psychology of this University.

### 2.2 Stimuli and procedure

All stimuli were presented on a computer screen with gray background situated 120 cm away from the participant. Psychopy software [3] was used to design the experiment and display the stimuli.

The design of the experiment is the same change detection task used in Vogel (2004) with variations in size and distances of the stimuli. At the beginning of each trial, a central arrow cue instructed the participants to remember the items in either the left or the right hemifield. Afterwards, a memory array appeared with the items (100 ms), which can be composed by one item (low memory load), two items (medium memory load) or four items (high memory load). After a retention period of 900 ms, a test array appeared in which the color of one square was different from the corresponding item in the memory array in 50% of trials; the colours of the two arrays were identical on the remaining trials. Participants had to report if color squares were identical or different between memory array and test array. The task was composed by 96 trials, with 32 trials for each condition of memory load (one, two and four items).

All stimulus arrays were presented within two 7.2° × 13.15° rectangular regions that were centered 5.4° to the left and right of a central fixation cross on a gray background. Each memory array consisted of 1, 2 or 4 colored squares (1.17° × 1.17°) in each hemifield. Each square was selected at random from a set of seven highly discriminable colours (red, blue, violet, green, yellow, black and white), and a given colour could appear no more than twice within an array. Stimulus positions were randomized on each trial, with the constraint that the distance between squares within a hemifield was at least 3.5° (centre to centre).

### 2.3 Behavioural data processing

Working memory capacity (WMC) was calculated using the formula developed by Pashler (1988). The formula is $\;WMC\;=S\;\times \left(\frac{H-F}{1-F}\right)$, where H is the observed hit rate, F the false alarm rate and S is the higher set size (maximum number of to-be-remembered items). The total accuracy was calculated as the hit rate for the whole experiment including all set sizes conditions.

### 2.4 EEG recording

EEG data was obtained using 64 electrodes (Biosemi ® ActiveTwo) arranged according to the international 10/20 extended system. Horizontal and vertical eye movements were monitored using four external electrodes. Horizontal EOG was recorded bipolarly from the outer canthi of both eyes and vertical EOG was recorded from above and below of the participant's right eye. Two additional external electrodes were placed on the right and left mastoid to be used for later re-referencing.

### 2.5 EEG data pre-processing

Data pre-processing was performed using Matlab R2018b (The Mathworks, Inc.) with EEGLAB 14_1_2b toolbox [2]. The signal was down-sampled off-line at 1024 Hz. Because of hardware setup constraints, all electrodes were referenced to CMS and DRL during acquisition, but off-line re-referenced to averaged mastoids.

The EEG signal was segmented in epochs of 1400 ms length, selecting the 200 ms preceding the memory array appearance up to 1200 ms after that (based on codes from Table 1). Baseline correction was carried out using the 200 ms time window preceding stimulus appearance. Only correct trials were analysed.

Muscular and movement artefacts detection was performed on epoched data by manual inspection, blind to condition. All epochs with artefacts were rejected. Independent component analysis (ICA) was performed using EEGLAB toolbox. Afterwards, the independent component associated with blinks was rejected for each participant.

### 2.6 ERP calculation

For ERP analysis, a 2nd order infinite impulse response (IIR) Butterworth filter was used for band-pass filtering continuous EEG data, with a half amplitude cut-off frequency of 0.01 Hz and 20 Hz. We calculated contralateral delay activity by averaging the activity recorded at right posterior electrode sites (TP8, CP6, CP4, CP2, P2, P4, P6, P8, P10, PO8, PO4, O2) when participants were cued to remember the left side of the memory array with the activity recorded from the left posterior electrode sites (TP7, CP5, CP3, CP1, P1, P3, P5, P7, P9, PO7, PO3, O1) when they were cued to remember the right side. Contralateral delay activity (CDA) was measured as the difference in mean amplitude between the ipsilateral and contralateral waveforms, with a measurement window of 300–900 ms after the onset of the memory array. CDA was calculated separately for memory array of one, two and four items.

### 2.7 Statistics

We calculated the difference in amplitude of CDA between two and four items, for each participant. Pearson correlation test was performed to assess correlation between the difference in CDA and WMC for each participant. This analysis was performed using SPSS software.

### 2.8 Behavioural and electrophysiological data

Participants performed the experiment with a total accuracy of 85.6 + 7% (mean + SD). The working memory capacity (WMC) was 2.3 + 0.8 (mean + SD).

We replicated the results from Vogel 2004, showing that the amplitude of the contralateral delay activity (CDA) is sensitive to the number of items in the memory array (Fig. 2, left). Specifically, the amplitude of CDA increase with the number of items that participants had to maintain in visual working memory. Furthermore, the increase in amplitude of CDA between two and four items was correlated with WMC calculated for each participant (r = −0.4485; p = 0.0159). We can observe in Fig. 2 (right) that participants with high WMC showed larger amplitude increase in CDA between two and four items, compared with participants with low WMC. We also observed this correlation between the magnitude of CDA increase between one and four items and WMC (r = −0.4606; p = 0.0135).
